# Editorial: New horizons in human bonds: insights into interpersonal synchrony across the lifespan

**DOI:** 10.3389/fpsyg.2026.1975365

**Published:** 2026-09-28

**Authors:** Anu Tourunen, Wolfgang Tschacher, Liam Cross, Virpi-Liisa Kykyri, Evrinomy Avdi

**Affiliations:** 1 Department of Psychology, Faculty of Education and Psychology, University of Jyväskylä, Jyväskylä, Finland; 2 Universitat Bern, Bern, Switzerland; 3 School of Psychology, University of Plymouth, Plymouth, United Kingdom; 4 School of Psychology, Aristotle University of Thessaloniki, Thessaloniki, Greece

**Keywords:** attunement, concordance, interpersonal coordination, interpersonal synchrony, linkage, mimicry, mirroring, responsiveness

Interpersonal synchrony refers to temporal coordination between individuals across different interactional modalities, such as behavior or nervous system activity. Interest in synchrony has grown substantially across research fields over the past decade. Synchrony has been associated with affiliation, empathy, cooperation, and effective interaction (Mogan et al., 2017; Rennung and Göritz, 2016), and in psychotherapy with alliance, outcome, and therapeutic change (Jennissen et al., 2025; Tourunen et al., 2022; Tschacher and Meier, 2020). However, synchrony has also been linked to diverse outcomes, including conformity and obedience to destructive acts (Cross et al., 2019).

Enough evidence has accumulated that it is no longer sufficient to ask simply whether synchrony exists or is beneficial. The field should examine what gives rise to synchrony, what follows from it, and the variables that moderate or mediate these relationships. Listening to a concert, dancing together, being in therapy, and having a daily conversation are not interchangeable interactional situations, and synchrony may emerge in one “channel” of interaction but not another. Furthermore, terminological distinctions remain inconsistent, particularly between coordination and synchrony, while anti-phase coupling and other forms of negative and complementary synchrony are often overlooked (Cross et al., 2016).

This Research Topic brings together relevant issues in interpersonal synchrony research in different study populations and highlights some of the conceptual and methodological challenges. The contributions examine how interpersonal synchrony is organized, what mechanisms sustain it, and under which conditions it matters.

## From correlation to causation

Associations between synchrony and social outcomes do not establish that synchrony itself produces those outcomes. Synchrony could instead be a byproduct of some other process, such as shared attention or prior liking, that independently drives both coupling and affiliation. Settling this requires manipulating synchrony directly and confirming that the manipulation worked.

Cross et al. addressed this challenge using virtual reality, manipulating and behaviorally verifying synchrony between participants and a virtual drumming partner. Synchronous drumming increased affiliation and, with a virtual partner presented as belonging to another ethnic group, also increased trust and reduced prejudice toward that group. The study demonstrates how synchrony can be treated as an experimental variable rather than merely inferred from an assigned condition.

## Timing and direction

Synchrony is not necessarily simultaneous, symmetrical, or constant throughout an interaction. Tschacher et al. examined the direction of electrodermal synchrony between patients and therapists. The therapeutic bond was stronger, and patients reported less session distress when changes in patient arousal were followed by similar changes in therapist arousal. Thus, the relational significance of physiological synchrony may depend on both the direction and development of synchrony over time.

Synchrony may also take time to emerge. Across repeated neurorehabilitation music therapy sessions, Yap et al. found above-chance heart-rate synchrony and an association between synchrony and intervention duration. Together, these studies frame synchrony as a temporally unfolding process whose meaning may depend on both its direction and development over time.

## Capacities to synchronize

The capacity to attune to others may vary across development and take different forms across individuals. In the group-play study by Nguyen et al., preschoolers attained similarly low levels of synchrony with partners assigned to interact synchronously or asynchronously. The authors suggest that the attentional and motor demands of maintaining a rhythm while distinguishing between two partners may have constrained the children's ability to synchronize in this group setting.

Attunement need not rely exclusively on observable bodily matching. Through phenomenological interviews, Zepeda et al. identified different forms of empathic engagement in people with Parkinson's disease, ranging from bodily-affective resonance to more visually and cognitively mediated engagement. Reduced bodily resonance did not necessarily imply an absence of emotional response.

Extending attunement to person–environment relations, Kumar proposes that autism-related mis-attunement may arise from differences in sensorimotor processing and in fit with eco-social environments. This theoretical model shifts attention from individual capacities alone toward compatibility between individuals and their environments.

Understanding these varied capacities and forms of attunement requires distinguishing relatively stable individual tendencies from context-dependent variation. Goldman et al. present a multimodal digital phenotyping framework for distinguishing stable, trait-like characteristics from changing, state-like processes. Applied to synchrony research, this distinction could help determine whether individuals have relatively stable synchrony “signatures” or whether coordination primarily reflects the particular relationship, task, and moment.

## Toward a more nuanced understanding of synchrony

Taken together, these contributions support a shift from treating synchrony as a general index of social connection toward examining its mechanisms, dynamics, and functions. Key questions concern how synchrony varies across individuals, partners, contexts, and modalities, and how the concept relates to empathy, liking, alliance, and co-regulation. Established relationships may facilitate synchrony but attempts to coordinate may also help build relationships.

Answering these questions requires moment-by-moment analyses of synchrony dynamics, inspections of leader–follower patterns and synchrony networks, and multimodal, mixed-method research. It also requires greater methodological transparency. Synchrony estimates depend on factors such as sensor placement, temporal alignment, preprocessing, analysis windows, and lags. Systematic sensitivity analyses and shared open datasets would help determine whether methods capture convergent or distinct properties of synchrony.

Interpersonal synchrony may thus be best understood as *a dynamic, relational, and multimodal process emerging from the interplay of individual characteristics, relationships, interactional tasks, and broader ecological contexts*. Its consequences may depend less on synchrony itself than on how coordination unfolds, in which direction, and for what regulatory function. The aim for future research is to develop precise, data-supported theories of how different forms of interpersonal coordination arise, change, and matter across relationships and the lifespan.
